# Cancer vaccines: from an immunology perspective

**DOI:** 10.1093/immadv/ltad030

**Published:** 2023-12-21

**Authors:** Shania Makker, Charlotte Galley, Clare L Bennett

**Affiliations:** Department of Haematology, UCL Cancer Institute, University College London, London, UK; Barts and the London School of Medicine and Dentistry, London, UK; Department of Haematology, UCL Cancer Institute, University College London, London, UK; Department of Haematology, UCL Cancer Institute, University College London, London, UK

**Keywords:** Immunotherapy, dendritic cells, mRNA vaccines, cancer vaccines

## Abstract

The concept of a therapeutic cancer vaccine to activate anti-tumour immunity pre-dates innovations in checkpoint blockade immunotherapies. However, vaccination strategies have yet to show the hoped-for successes in patients, and unanswered questions regarding the underlying immunological mechanisms behind cancer vaccines have hampered translation to clinical practice. Recent advances in our understanding of the potential of tumour mutational burden and neo-antigen-reactive T cells for response to immunotherapy have re-ignited enthusiasm for cancer vaccination strategies, coupled with the development of novel mRNA-based vaccines following successes in prevention of COVID-19. Here we summarise current developments in cancer vaccines and discuss how advances in our comprehension of the cellular interplay in immunotherapy-responsive tumours may inform better design of therapeutic cancer vaccines, with a focus on the role of dendritic cells as the orchestrators of anti-tumour immunity. The increasing number of clinical trials and research being funnelled into cancer vaccines has demonstrated the ‘proof-of-principle’, supporting the hypothesis that therapeutic vaccines have potential as an immuno-oncology agent. For efficacious and safe cancer vaccines to be developed, better understanding of the underpinning immunological mechanisms is paramount.

## Introduction

Vaccination of cancer patients to elicit anti-tumour immunity and long-term protection against relapse has long been a vision for immune oncologists. The concept that our immune system could be activated to detect and destroy malignant cells has been embedded in the field since Coley’s early treatment of cancer patients with bacterial lysates [1] and the formulation of the theory of immune surveillance by Burnet [2]. Subsequent identification of tumour-specific antigens (TSAs) reinforced the concept that tumours could be recognised by T cells as an altered-self, and this would be sufficient for cancer elimination. The discovery of dendritic cells (DCs) as ‘nature’s adjuvant’ [3] provided a platform for tumour antigen delivery and further fuelled the expansion of the cancer vaccine field. But, despite this long-held belief that therapeutic vaccines could contribute to cancer treatment, a lack of convincing beneficial clinical outcomes in patients has hampered the field.

Immuno-oncology has revolutionised the field of cancer therapeutics leading to dramatic improvements in survival in some patients, largely due to the use of immune checkpoint inhibitors (ICIs). Detailed analysis of T cell responses in those patients that respond to ICIs has revealed therapeutically active tumour-reactive T cells that are sufficient to initiate tumour rejection [4], re-igniting interest in the development of cancer vaccines at a time when global infectious disease challenges have driven vaccination technologies. In 2023, the UK government announced a partnership with BioNTech to use advances in mRNA vaccine technology for the treatment of cancer patients; the aim being to develop personalised mRNA vaccines encoding patient-specific antigens for treatment of a range of cancers [5]. Critically, this partnership benefits from the NHS/Genomics England Cancer Vaccine Launch Pad which will serve to identify and recruit patients undergoing surgery who may benefit from this approach [6].

Despite these advances, for therapeutic cancer vaccines to be successful we need a better understanding of how they work and when to use them. Unlike traditional preventative vaccines, therapeutic delivery of cancer vaccines depends on immune activation within a diseased environment. While some dogmas of immunobiology from infectious diseases will remain we need to better understand how cellular mechanisms are altered in cancer patients. A key example is our evolving understanding of the roles DCs play in anti-tumour immunity; recent data highlight new functions for intra-tumoural DCs that were not previously revealed using other disease models [7]. In addition, our perspective has changed with the advent of ICIs. Traditionally, vaccination as a monotherapy was a route to priming naïve T cells but, despite numerous clinical trials over the years, we have yet to see much clinical benefit with this approach (illustrated in Table 1). Rather, focus has shifted to use of vaccines as combinatorial treatments with ICIs and/or other approaches such as chemo- and radiotherapy. In these contexts, vaccination becomes a tool to augment T cells in patients who lack pre-existing tumour-reactive cells, to push primed T cells towards responsiveness in the tumour microenvironment (TME) and bolster those T cells that have not responded to immunotherapy. Defining the therapeutic context for vaccination will determine how vaccines regimens are designed. Here, we focus on our growing understanding of DC immunobiology in tumours to discuss evolving approaches to therapeutic cancer vaccines and the development of personalised mRNA vaccines.

**Table 1.** A snapshot of therapeutic cancer vaccine trials and their clinical and immunological outcome

Trial phase	Cancer	Vaccine	Immunological responses	Clinical outcome	Reference
I	Non-small cell lung cancer	Vaccination with 4 HLA-A24-restricted peptides.	87% of patients with T cell responses to at least one of the peptides.	47% of patients had 2 months of stable disease with no complete responses.	[8]
	Non-small cell lung cancer	MAGE-A3 vaccine with standard chemotherapy treatment.	CD4+ T cell response 36%, 27%, 25%, and 83% in different cohorts and in 4 patients CD8 T cell responses were also detected.	Grade 3 adverse events were recorded for all cohorts but normal for chemotherapy.	[9]
	Non-small cell lung cancer	Personalised neoantigen vaccine NEO-PV-01 with standard first-line treatments for non-small cell lung cancer.	Both CD4+ and CD8+ T cell responses in all patients.	Did not observe patient outcomes.	[10]
	Pancreatic ductal adenocarcinoma (PDAC)	Individualised RNA neoantigen vaccine with anti-PD-L1.	50% of patients with antigen-specific T cell responses.	Extended median-recurrence free survival for 50% of patients with the prolonged T cell response.	[11]
	Melanoma	FixVac (BNT111), Lipo-Merit RNA-LPX.	Polyclonal CD4+ and C8+ T cell responses detected.	Durable clinical responses were observed.	[12]
	Melanoma	Personalised DC cellular vaccines.	Detected HLA class I restricted specific antigens with range of T cell receptor collection.	Did not observe patient outcomes.	[13]
	Ovarian cancer	Tumour-pulsed monocyte-derived DC vaccine with expanded T cell populations.	Novel antigen-specific peptides were observed in patients.	Showed clinical improvements but did not eliminate tumours all together.	[14]
I/II	Gastrointestinal	Personalised mRNA vaccine.	Antigen-specific CD8+ and CD4+ T cell responses detected.	No clinical responses were seen in 75% of patients.	[15]
	Prostate	BNT112, RNA-LPX vaccine encoding for 5 TAAs.	All patients had immune responses induced by the vaccine with responses to all 5 antigens in 40% of patients.	PSA levels were reduced in 2 of the patients.	[16]
	Breast	DCs pulsed with HER2 peptides, administered with anti-HER2 antibody.	Increased T cell clonal expansion in group of patients but immune response did not correlate to clinical outcome.	Stabilised diseases state for 46% of patients, small number of patients with partial response.	[17]
II	Non-small cell lung cancer	MUC1 with chemotherapy treatment.	In-depth immunological responses were not analysed.	Median progression-free survival was 5.9 months for the administered vaccine group and 5.1 for placebo group and the primary endpoint was met.	[18]
	Non-small cell lung cancer	Vx-001 targeting TERT.	Long-lasting TERT-specific immune response with increased specific CD8+ T cell response.	Failed to meet their primary endpoint. Over 29% of responders had increased OS (overall survival) compared to non-responders.	[13]
	Melanoma	Fusion anti-DEC-205-NY-ESO-1 antibody with Flt3-L treatment.	Significant increase in both B cell and T cell responses, with increased long-term immunity of both CD4+ and CD8+ T cells.	Did not assess the therapeutic outcome, end point was measured through immune response.	[19]
	Breast cancer	AE37 peptide vaccine.	In depth immunological responses were not analysed.	No change in disease free survival with vaccine administered.	[20]
	Glioblastoma multiforme (GBM)	Audencel, DC cellular vaccine.	Increased production of IFNγ and interleukin 2 with increased numbers of CD8+ T cells.	Overall failed see any clinical improvements.	[21]
	Esophaheal cancer	HLA-A*24-restricted tumour antigen epitope as a peptide vaccine.	Increased CD8+ T cell responses.	Decreased recurrence rate but no increase in relapse free survival.	[22]
III	Non-small cell lung cancer	START study: Tecemotide, peptide vaccine.	In depth immunological responses were not analysed.	No overall survival difference observed.	[23]
	Non-small cell lung cancer	MAGRIT study: MAGE-A3, cancer testis antigen.	In depth immunological responses were not analysed.	No overall survival difference observed.	[24]
	Metastatic melanoma	Gp100 peptide vaccine with ipiliumab.	In-depth immunological responses were not analysed.	Gp100 alone did not had reduced OS compared to ipilimumab alone, or in combination.	[25]
	Glioblastoma	Rindopepimut peptide vaccine.	In-depth immunological responses were not analysed.	No overall survival was seen.	[26]

## The immunology of tumour rejection

### DC function in tumours

Curative anti-tumour T cell immunity has been conceptualised in the framework of the cancer-immunity cycle whereby intra-tumoural DCs capture antigens, migrate to draining LNs, and prime naïve T cells that migrate back to tumours, kill antigen-specific cells, and release more antigens to perpetuate the cycle [27]. As such, DCs are critically positioned as vital cells for the activation of a durable, protective T-cell response. Conventional (c)DCs can be classified into two developmentally distinct subsets: cDC1s efficiently cross-present exogenous antigens on MHC I and secrete IL-12, making them potent stimulators of CD8^+^ cytotoxic T cells and Th1 cells, while cDC2s may be more important for presentation of MHC-II-peptide complexes to CD4^+^ Th2 and Th17 cells [28]. Importantly, these populations are highly conserved between mice and humans [29]. cDC1s are required and sufficient for anti-tumour immunity [30, 31], transporting tumour antigens to draining LNs to prime T cell responses [32]. The importance of this rare population of cells is evidenced by the extensive measures adopted by tumours to exclude cDC1s [33–36], with lower levels of cDC1s seen in tumoral compared to healthy tissue [37]. A positive correlation between cDC1 numbers in tumours and patient survival further highlights the critical importance of these cells for anti-cancer immunity [38, 39]. Unlike cDC1s, which are a relatively homogeneous population of cells [29,40], cDC2s are significantly more heterogenous, revealed through single-cell sequencing of patient tumours which demonstrates overlap, at the transcriptional level at least, with non-conventional monocyte-derived DCs [40]. cDC2s prime CD4^+^ T cell-mediated anti-tumour immunity when released from suppression by tumour regulatory T cells [41] and stimulation with type 1 interferons promotes cDC1-like cross-presentation to CD8^+^ T cells from cDC2s [42]. These interferon-stimulate gene^+^ cDC2s may therefore have therapeutic potential, but we still lack understanding of how cDC2 sub-populations are related and their relative contribution to or against anti-tumour immunity; indeed one study using elegant combinations of genetically modified mice has suggested that, in fact, cDC1 are competent to prime both CD4 and CD8 T cells in tumours [43].

While the cancer immunity cycle focuses attention on the importance of DCs to prime naïve tumour-reactive T cells, there is an established body of evidence demonstrating the critical role of intra-tumoural DCs as orchestrators of effector/memory T cell immunity. Production of IL-12 by tumour DCs is required for responsiveness to chemotherapy and ICIs [44] [45], and intra-tumoural CCR7^neg^ cDC1s recruit stem cell-like TCF1^+^ CD8 T cells expressing CXCR3 to form clusters of cells that correlate with improved patient survival [46]. In addition, intra-tumoural CCR7^+^ DCs, that remain within the TME despite expression of the classic LN-homing chemokine receptor CCR7, may be important, although the precise relationship of these cells to other DC populations is still being discussed [47]. CCR7^+^ DCs cluster around tumour blood vessels and both recruit cytotoxic T cells via production of CXCL16 and ensure their survival due to trans-presentation of IL-15 [48]. This previously unappreciated role for DCs within the TME could explain the conundrum of how such a rare population of cells can dominate anti-tumour immunity, concentrating T cell activation and survival within specific intra-tumoural niches; formation of DC-CD4-CD8 ‘triads’ promoted restoration of exhausted T cells and response to ICI [49]. Targeting DCs to enhance formation of these clusters therefore has exciting therapeutic implications.

### Tumour-reactive T cells

The importance of CD4 and CD8 T cells for anti-tumour immunity is well-defined and extensively reviewed elsewhere. Increasing attention is focussed on a sub-population of TCF1^+^CD8^+^ T cells (also referred to as ‘precursors of exhausted T cells’ (Tpex)) that preserve the effector CD8 T cell compartment under conditions of chronic antigen stimulation and exhaustion in tumours. The transcription factor TCF1, encoded by the gene *TCF7*, is required for formation of a pool of renewable central memory T cells during viral infections [50]. Upon stimulation, TCF1^hi^ cells self-renew to maintain a TCF1^hi^ T cell pool but also produce TCF1^low^ CD8^+^ effector T cells, suggesting that TCF1 is a critical contributor to the stem-like properties of these cells [51]. TCF1^+^CD8^+^ T cells exhibit an ‘exhausted’ phenotype, expressing high levels of inhibitory receptors such as PD-1 and Lag-3 in chronic viral infections and tumours [52,53] and are therefore key targets for ICIs, which both mobilise TCF1^+^CD8^+^ T cells and activate differentiation into effector T cells for tumour rejection [53–55]. These findings suggest a possible mechanism for the therapeutic benefit of ICIs, whereby the induction of stem-like properties as well as effector T cells is critical in the anti-tumour response [55].

Whether therapeutic vaccines need to target T cells released from the exhausted TCF1^+^CD8^+^ pool or synergise with ICIs by co-expanding other T cells remains to be tested. cDC1s provide MHCI-dependent lymphoid organ niches for Tpex during viral infection [56] and a similar model has been proposed in LNs of mice bearing lung tumours [57]. In the latter case, provision of the DC growth factor Flt3L with agonistic anti-CD40 to increase the frequency of activated migratory cDC1s in LNs was sufficient to enhance TCF1^+^CD8^+^ T cell numbers and reduce tumour burden [57]. By comparison, evidence that CCR7^neg^ cDC1 present antigen to TCF1^+^CD8^+^ T cells within tumours [46], makes this interaction an attractive vaccine target.

## Choosing tumour antigens and the development of personalised vaccines

The guiding principle of cancer vaccination is the concentrated direct or indirect delivery of antigens to DCs to facilitate presentation to T cells. The choice of antigen in a therapeutic cancer vaccine is therefore of critical importance; ideally tumour-specific and sufficiently different to elicit a functional T cell response from non-tolerised T cells [58, 59]. Tumour associated antigens (TAA) such as gp100 or MUC1, that are tissue- but not tumour-, specific were originally attractive targets due to the broad therapeutic usage across patients, but central tolerance to these antigens limits the efficacy of these vaccines as only T cells with low antigen avidity may be available for activation; however, evidence suggests that even some of these TAAs such as MUC1 may be differentially glycosylated between normal and tumour tissue, suggesting potential therapeutic benefit if targeted [60]. However, toxicities due to the induction of T cell responses against shared antigens in healthy tissues largely preclude the use of TAAs. The cancer-testis antigens are expressed during development, epigenetically silenced in healthy tissue, but frequently re-expressed in malignant cells. As such, antigens like NY-ESO-1 may be considered TSAs and have been widely used as model vaccine antigens. In addition, vaccines targeting mutated self-antigens such as KRAS^G12D^ [61] and mutated anaplastic lymphoma kinase (ALK) have had some success, e.g. in patients with ALK-rearranged lung cancer [62]. Recent advances in sequencing technologies have expanded the repertoire of unique antigens revealing tumour-specific mutated genes. These so-called ‘neo-antigens’ may arise from non-synonymous somatic mutations in the coding region of endogenous genes, insertion or deletion events, gene fusions, translocations, and splice variants, as reviewed in [59], but also from integrated retrotransposons [60] or even bacteria [63].

**Figure 1.**
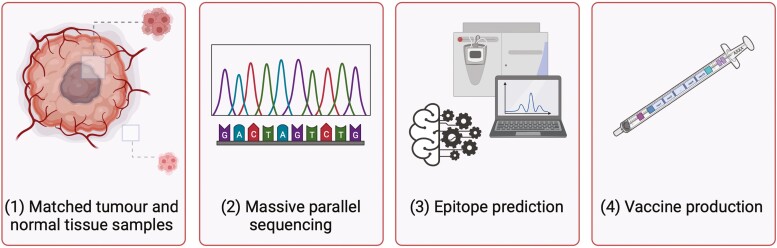
Neo-antigen vaccine development process. Matched samples are obtained from the tumour and normal tissue (1) and processed by massive parallel sequencing to obtain the DNA sequences for the tumour and normal tissues (2). These samples can then be aligned to the reference genome to identify somatic mutations, i.e. differences in the DNA sequence between the reference genome and tumour tissue, compared to the differences seen in the DNA sequence between the reference genome and normal tissue. Based on these somatic mutation calls computational algorithms predict epitopes (3). This information can be used to design and produce neo-antigen vaccines which are personalised to each patient. Created with BioRender.com.

With the advent of next-generation sequencing, it has been possible to identify neo-antigens that are specific to each patient, thus allowing the possibility for personalised vaccines [64]. Matched non-malignant and tumour biopsies from a patient are processed for massive parallel sequencing to compare DNA sequences between normal and tumour tissue [65], and both the normal and tumour samples are aligned to a reference genome. Somatic mutations are called by identifying differences between the reference and the tumour that are not different between the reference and normal tissue [65]. The next step is epitope prediction using various algorithms to predict the binding affinity of predicted antigens to MHC, considering factors such as antigen processing and epitope abundance [66]. Epitope abundance can be classified as present or absent using DNA sequencing or quantified using RNAseq to understand expression levels [11,15]. Neo-antigen(s) of choice are encoded in a vaccine vector and used to immunise the patient (Fig. 1). Early proof-of-principle experiments combined the use of exome sequencing and mass spectrometry to predict neo-antigens in the murine tumour lines TrampC1 and MC38. These data demonstrated feasibility of identification of MHCI-binding peptides derived from tumour-specific mutations and generation of therapeutic vaccines in mouse models [67]. This principle was validated in early clinical trials by the Linette lab [13], who used exome sequencing of resected stage 3 melanomas with *in silico* modelling to identify potential HLA*02-binding mutated peptides. Vaccination with peptide-pulsed autologous, matured DCs resulted in both expansion of neo-antigen-specific T cells, and activation of T cells specific for sub-dominant neo-antigens compared to T cells pre-vaccination, suggesting that vaccination had increased the breadth of the T cell response. These patients had previously received ICIs, and overall response data was not collated, so the clinical impact of vaccination is yet to be determined.

A huge array of algorithms have been developed to-date to enhance epitope prediction of MHC-I- and II-binding epitopes [66, 68, 69], and are constantly being refined [70]. Understanding the binding affinity may help to envisage the degree of anti-tumour immune response, with efforts focussed on identifying neo-antigens with strong binding affinities [71]. But, while there has been some success in predicting MHC-I epitopes, prediction of MHC-II peptides has proved more challenging due to structural differences that allow for greater variety in length and location of binding antigens [68]; advances in the field were made with the development of the algorithm NetMHCIIpan [72]. Use of artificial neural networks and deep learning models may further enhance neoantigen prediction [69].

Studies associating tumour mutational burden (TMB) with response to ICIs [73–75] have fuelled the use of neo-antigens in cancer vaccine development. But this correlation does not necessarily hold true across all cancers and recent analysis of large-scale ‘pan-cancer aggregate’ data suggested that, when confounding factors are removed, TMB is in fact a poor predictor of ICI response and survival [76]. In addition, the nature of the TMB may be important; persistent mutations in single-copy regions that are less easily lost under selective immune pressure were more likely to be correlated to response to ICI [77]. It is likely that only some non-synonymous mutations will lead to the generation of novel immunogenic peptides, and it may not be the burden of neo-antigens within a tumour, but the immunogenicity of the neo-antigen, which is vital in generating a strong anti-tumour immune response [76, 78]. Clonal heterogeneity within tumours will also affect the relative importance of the TMB and it has been shown that clonal TMB was a stronger predictor of response to ICI than sub-clonal TMB [79]. Using an elegant panel of genetically mutated mice to generate DNA mis-matched repair deficient (MMRd) tumours, Jaeger *et al.* demonstrated that sporadic MMRd (and consequent increased TMB) *per se* was not sufficient to elicit tumour immunogenicity. Importantly, post-translational modifications impacted evolution of the immunopeptidome, shown by the increase in number of MHC-I peptides identified subsequent to HSP90 inhibition [80]. Together these findings demonstrate that we still need to understand more about the evolution of TMB in the context of immune selection to determine which neo-antigens will be beneficial immunotherapy targets.

## Cellular platforms for cancer vaccines

There is still little consensus on the optimal platform for the delivery of cancer vaccines. Mirroring preventative vaccination strategies with live attenuated or killed viruses, the use of cell-based vaccines has been extensively tested, with a focus on the use of patient DCs as the cellular product. Alternatively, platforms that permit vaccination with tumour antigens and adjuvants seek to engage endogenous DC populations at the injection site. These different approaches have been extensively reviewed elsewhere (e.g. [81]).

Injection of autologous whole tumour cells provides a simple, direct method of providing the full spectrum of tumour antigens. This array of proteins contains epitopes for CD4+ and CD8+ T cells, facilitating MHC classes I and II presentation, and promoting a broad anti-tumour immune response that may reduce chances of tumour escape and development of resistance [82, 83]. Activation of CD4+ helper cells aids continuous T cell activation, clonal expansion, and differentiation of CD8+ effector and memory cells, improving durability of the immune response. Use of dead cells enhances immunogenicity and reduces secretion of soluble factors by the vaccine product which may interfere with the immune response [83], while other recent technologies include the use of photothermal nanoparticles which can be activated to induce heat shock proteins as endogenous adjuvants [84]. Use of allogeneic tumour cells expanded from manipulated cell lines or tumour biopsies to provide more generic, relatively low-cost vaccines has been explored [72]. This field has been led by the use of GVAX, an irradiated melanoma cell line that expresses the DC growth factor GM-CSF [81]. More recently, in an innovative approach, Chen *et al*. engineered a murine glioblastoma cell line to secrete IFNβ and GM-CSF, while removing sensitivity to IFNβ-induced cell death by deletion of *Ifnar1*, the IFNβ receptor [85]. Implantation of these cells in the brain cavity after resection of primary tumours prevented recurrence of disease, and treatment was associated with accumulation of CD11c^+^MHCII^+^ DCs at the tumour site.

The key disadvantage to a cellular vaccine approach is the low concentration of immunogenic antigen within the product. To circumvent this many labs have focussed on the use of autologous DCs as a platform to inject concentrated tumour antigens. One of the first DC cellular vaccine trials, preceding ICIs by more than a decade, was performed in metastatic melanoma patients [86]. Monocyte-derived DCs generated in GM-CSF/IL-4 cultures were pulsed with tumour lysate or specific peptides and injected into 16 patients, of whom 12.5% demonstrated a complete response to the treatment. This success in the absence of significant toxicities, reinforced the concept of DCs as the platform for cellular vaccines.

The Sipuleucel-T (Provenge®, Dendreon Pharmaceuticals LLC) vaccine for metastatic castration resistant prostate cancer (mCRPC) is the first and only licensed DC based cancer vaccine; when announced, it was a cornerstone for immunotherapy and an exciting new prospect in treating patients for whom there were no other options. Patient peripheral blood mononuclear cells were cultured with the fusion protein PAP2024, consisting of GM-CSF and the tumour-related protein, prostatic acid phosphatase (PAP). The product was injected back into patients, wherein it was assumed that activated antigen presenting cells (APCs) presenting PAP antigens would migrate to LNs and prime anti-tumour CD8 T cell immunity [87]. In the initial phase III IMPACT trial, Sipuleucel-T resulted in a small but significant prolonged overall survival of 4 months [87]. But, there was no observed reduction of tumour burden and numerous subsequent clinical trials since have produced conflicting data (for a summary see [88]). This confusion persists today, e.g. a recent clinical trial testing administration of Sipuleucel-T with radium-223, a radioactive element used in prostate cancer therapies, recorded reduced PSA levels in patients and longer overall survival but observed a reduction in anti-tumour immune responses [89]. We argue that this confusion partly arises partly from the fact that we still do not fully understand the mechanisms by which Sipuleucel-T may activate anti-tumour immunity. Despite frequent descriptions of Sipuleucel-T as the original DC vaccine, there is, to our knowledge, little evidence that activation of DCs is central to any therapeutic effect from the vaccine. Antigen-specific T cell responses were observed in most vaccinated patients [90] and other studies have demonstrated increased CD8 T cell infiltration into tumours [91, 92]. But, a more detailed characterisation of peripheral blood samples in a subset of patients under the IMPACT trial reported that the injected product contained a median of 18.3%APCs the majority of which were CD14+ suggesting a monocytic origin [90]. This was based on the use of CD54 (ICAM-1) as a marker to both identify APCs and track activation [93] rather than more established phenotyping markers (e.g. CD11c, HLA-DR). The authors did, however, observe production of IL-12p70 in Sipuleucel cultures, potentially suggesting activation of monocytes and monocyte-derived DCs.

We now understand that the original GM-CSF/IL-4-generated monocyte-derived (mo)DCs were unlikely to be potent stimulators of T cells *in vivo*; differentiation *in vitro* does not mimic physiological pathways, resulting in cells that are less efficient at activating T cells than their *in vivo* counterparts, and which, contrary to original expectations, do not migrate to draining LNs [94]. A classic study in which radio-labelled moDCs were injected into healthy volunteers demonstrated that the majority of cells remained at the injection site [95]. More recent approaches to overcome this caveat include electroporation of moDCs with a mix of co-stimulatory and activating factors (TriMix: CD40L, CD70, constitutively active TLR4) [96, 97], or co-injection of cells expressing GMCSF, CD40L, and CCL21 to enhance migration of activated local DCs to LNs [98]. There may be value in using more physiologically relevant DC populations, but this approach carries the caveat that primary blood-derived DCs may be metabolically skewed within cancer patients resulting in suboptimal vaccine products [99]. To by-pass this, cDC1 can be differentiated from CD34^+^ stem cells [7], common DC precursors [100] or iPSC-derived DCs [101, 102]; in one recent advance, Makino *et al*., exploited Notch signalling to generate large numbers of cDC1s from iPSCs [103]. Other alternatives are so-called intelligent DCs, developed using outer membrane of mature DCs to coat nanoparticles. In murine studies, these artificial DCs migrated to draining LNs and presented antigens to T cells [103].

The short half-life of DCs [104] and poor migration upon injection led to suggestions that moDC vaccines may function as antigen deposits for endogenous DCs; the anti-tumour response to a moDC vaccine was abolished in Irf8+32^−/−^mice that selectively lack cDC1s [105]. By contrast, direct injection of cDC1 into tumours was sufficient to induce an anti-tumour response without engagement of endogenous cells in this model [105] suggesting that generation of cDC1 vaccines could overcome some limitations of moDCs. Together, however, these findings have prompted a shift towards engaging DCs *in vivo*.

## Cell-free platforms—exploiting endogenous DCs.

Vaccination with defined tumour peptides or proteins, or vectors encoding these genes, has the potential to deliver immunogenic antigens to tissue DCs, facilitating activation and priming of a T cell response. The numerous clinical trials that have been conducted using these approaches are detailed in other comprehensive reviews [64].

Peptide-based vaccines are composed of predicted or known tumour antigen epitopes, which may be short peptides or synthetic long peptides (SLP). Short peptides do not require processing by APCs and are directly loaded onto MHC-I molecules and presented to CD8+ T cells [106]. As such, short peptides may be presented to T cells in the absence of co-stimulation. In contrast, processing and presentation of SLPs permit a more sustained and effective immune response [107]. Adjuvants, such as synthetic toll-like receptor (TLR) ligands, and peptides such as PADRE to engage T cell help, are required to boost the immune responses [108]. Alternatively, use of vectors encoding nucleic acids (DNA or mRNA) exploits *in vivo* transcription and translation to produce tumour-specific proteins on site. DNA vaccines are relatively simple to produce and can encode many full-length tumour antigens, facilitating broader antigen presentation by transfected APCs [109]. DNA is more stable and persists longer than mRNA [110], and a single plasmid DNA can produce multiple mRNA copies and therefore antigens, in comparison to mRNA vaccines which generally contain one mRNA molecule, thus producing only one antigen [109]. However, DNA vaccines have relatively low immunogenicity, especially in comparison to mRNA vaccines, potentially due to the extra step of transcription which involves entering the cell nucleus [82]. Another possible limitation is the potential for DNA vaccines to integrate into the host genome, although the risk of insertional mutations is lower than that of spontaneous mutations [82]. Since the advent of mRNA vaccines for SARS-Cov2, these have somewhat superseded the use of DNA vaccines, as discussed in more detail below.

The efficacy with which DCs take up and process exogenous antigens is partly attributable to the range of endocytic/phagocytic receptors expressed by different DCs, including C-type-lectin receptors [111]. Therefore, it was proposed that targeting antigens to these receptors should enhance the loading of, and presentation by, tumour DCs. This approach was pioneered by Ralph Steinman in the early 2000s, who directly conjugated model antigens to antibodies targeting the C-type lectin receptor DEC205 [112, 113], and has subsequently been tested in patients. Combining an NY-ESO-1-DEC-205 vaccine (CDX-1401) with the adjuvant PolyI:C and Flt3L led to expansion of antigen-specific T cells and increased antibody titres in the blood of melanoma patients [113]; analysis demonstrated the expansion and activation of DCs [114]. While this trial was not sufficiently powered to detect differences in clinical responses, a previous clinical trial combing CDX-1401 with TLR adjuvants reported some stabilisation of disease, with the hint that this could be augmented with ICI [115]. To-date numerous other studies have tested different DC receptors, including Clec4a, Clec7a, and CD207/Langerin [28], but without progression to the clinic. It is notable that inclusion of Flt3-L with DC vaccination enhanced immune responses, suggesting that it may be necessary to increase DC abundance for full vaccine efficacy [19, 116]. Indeed, murine studies suggest that augmenting DC numbers alone is sufficient to activate anti-tumour immunity [34, 117]. However, with our growing understanding of the dominant suppressive nature of the TME is an awareness that, in addition to increasing DCs numbers, we may need strategies to directly remodel the local tumour environment to enhance DC activation and metabolism [99, 118]. An exciting new perspective has arisen from work by the Reis e Sousa lab demonstrating that tumours exploit the systemic actin scavenging system to prevent cross-presentation of tumour antigens by DCs via DNGR1/Clec9a [36]. We may need to release DCs from restraints such as these before we can fully benefit from their T cell-activating functions within patients.

## A new era of mRNA vaccines

First attempts at inducing gene therapy in 1990 by injecting genetic material into muscle cells showed great promise but also induced high immunogenicity and instability [119]. Thirty years on, and one global pandemic later, researchers have now turned their focus to developing mRNA vaccines for cancer therapeutics. The use of mRNA vaccines originally faced two major hurdles however, not encountered with other antigen-expression platforms: the first is the inherent lability of mRNA outside the nucleus, how to protect the message for long enough that it would be translated within the transfected cell? The second is the immunogenicity of cytosolic mRNA. Our innate immune cells are specialised at spotting and reacting to nucleic acids that are out of place and may indicate viral infection. An overabundant innate immune response may lead to vaccine-associated toxicities.

Packaging into lipid nanoparticles (LNPs) has provided a safe and effective way of delivering mRNA to cells, demonstrated by the success of this technology for prevention of COVID-19. Development of mRNA vaccines in cancer has focussed on optimising transfection and translation in DCs. Formulation of LNPs provides scope to manipulate cellular uptake and persistence, potentially by DCs [120, 121]. In addition, the length and modification of the poly(A) tail and 3ʹ UTR is a major determinant of translation efficiency in DCs [122, 123], and encoding mRNA may be further modified to enhance loading onto MHCI molecules [124]. Formulation of liposomes by increased concentration of cationic lipids produces lipoplex nanoparticles (mRNA-LPX) [125] that are preferred in clinical trials due to potency protecting RNA and the efficient uptake by DCs. Other advances have focussed on modifying mRNA structures to generate persistent responses via self-amplifying or trans-amplifying mRNA [126, 127], thus theoretically generating a larger antigen pool; there was no advantage between self-amplifying and non-amplifying mRNA when directly compared in an HSV-associated murine tumour model, but both performed better than protein or DNA vaccines [128]. The route of delivery is also important for targeting DCs; uptake of the SARS-CoV2 vaccine by DCs after intra-muscular (i.m.) injection is inferred in reviews discussing mechanisms of action [129, 130], but we believe that the site where this occurs remains opaque. Based on the assumption that i.m. injection will not reach a large number of DCs [131] the immune-oncology field has focussed on intra-venous injection of mRNA to enhance delivery to DCs and T cell activation [120, 125]. However, one open question is whether mRNA vaccines need to directly target DCs, or whether uptake and cross-presentation of (secreted) proteins from other transfected cells may provide a more durable, immunogenic response.

Solving the problem of immunogenicity has been initially credited to the work by Karikó and Weissmann [132, 133], recognised as recipients of the Nobel prize for Physiology and Medicine in 2023. They demonstrated that modification of RNA, e.g. by methylation, masked toll-like receptor (TLR)-dependent recognition by DCs [132]. This finding paved the way to generating more effective mRNA vaccines; incorporation of pseudouridine nucleotides not only prevents innate immune activation but enhances target protein expression [134]. However, these complexes may still activate the NLRP3 inflammasome and induce production of IL-1 cytokines that can cause inflammatory responses in some patients [135]. But some immunogenicity may be helpful; Kranz *et al*., demonstrated the requirement for RNA-LPX-induced interferon-alpha (IFN-ɑ) for activation of anti-tumour T cells [125].

The most promising trial data so far is from the BioNtech Lipo-MERIT trial in which patients with advanced melanoma were given 8 RNA-LPX vaccinations with a vaccine encoding four classic melanoma tumour antigens (NY-ESO-1, Tyrosinase, MAGE-A3, and TPTE) with or without anti-PD1 [12]. The interim analysis reported in 2019 demonstrated clear serum cytokine spikes (IL-6, IL-12p70, and IP-10) increasing with vaccine dose and expansion of antigen-reactive T cells *ex vivo*. Partial responses were noted in some patients, indicated by transient tumour regression, while, perhaps more significantly, two patients showed some tumour regression after failure to respond to ICIs, and, upon subsequent relapse, response to repeated treatment with anti-PD1 [12]. These data demonstrate that there may be some benefit from mRNA cancer vaccines that break tolerance to existing TAAs in the context of ICI. Extending these findings, preliminary data from the Moderna/Merck KEYNOTE-942 randomised phase 2 clinical trial have recently been reported (https://www.aacr.org/about-the-aacr/newsroom/news-releases/adding-a-personalized-mrna-cancer-vaccine-to-immunotherapy-may-prolong-recurrence-free-survival-in-patients-with-high-risk-melanoma/). Vaccination of melanoma patients with an mRNA vector encoding up to 34 personalised neo-antigens in combination with anti-PD1 prolonged recurrence-free survival compared to patients receiving ICI alone. These early data suggest that clinical benefit was independent of TMB, suggesting that provision of sufficient neo-antigens may overcome endogenous limitations due to lack of mutational burden, but follow up trials with more patients are needed to confirm these findings. A personalised mRNA-LPX vaccine has also been tested in pancreatic cancer patients [11]. Following surgery, patients received anti-PD-1, a personalised vaccine encoding 20 predicted neo-antigens and chemotherapy. The vaccine resulted in the robust expansion of polyfunctional T cell clones, associated with prolonged recurrence-free survival, compared to patients that had a combination chemotherapy treatment. Clinical trials using similar pipelines in patients with metastatic gastrointestinal cancer, but without ICIs, also demonstrated activation of antigen-specific T cells, but in the absence of clinical responses [15] suggesting that combination therapies are needed to push vaccine efficacy towards tumour rejection or prevention of recurrence. The PRO-MERIT trial has been set up to test an RNA-LPX vaccine for mCRPC that targeting 5 TAAs. Interim results indicate that prostate cancer patients that have received the RNA-LPX as a monotherapy have reduced PSA levels with some activation of TSAs [16].

## Closing remarks

**Figure 2.**
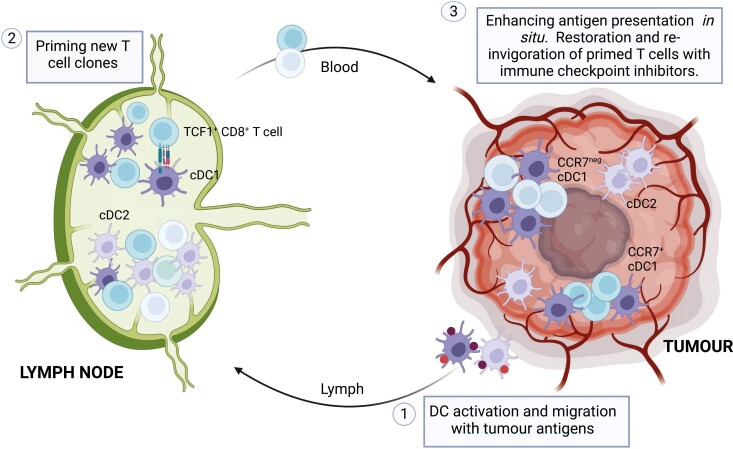
Targeting DCs to enhance therapeutic cancer vaccines. Our increased understanding of the role of DCs in anti-tumour immunity may inform opportunities for intervention: (1) vaccine-mediated delivery of antigens to DCs and activation of migration to LNs; (2) enhanced priming of naïve tumour-reactive T cells. Antigen presentation to Tcf1^+^CD8^+^ T cells may augment the pool of circulating effector T cells that are sensitive to immune checkpoint inhibition; (3) interventions that directly increase intra-tumoural DC activity (frequency, antigen presentation, activation) to re-invigorate exhausted T cells, probably in the presence of checkpoint inhibition. Created with BioRender.com.

Despite decades of unconvincing results, the convergence of our improving understanding of the immune responses that distinguish those patients who respond to immunotherapy, with advances in vaccine technologies, has re-ignited interest in the use therapeutic vaccines to treat cancer patients. Clinical trial data is offering a glimpse of clinical benefit in patients receiving personalised mRNA vaccines with ICIs, supporting the notion that cancer vaccines may find their value as immune-modulatory therapies, complementing other interventions rather than priming an anti-tumour T cell response *per se* (Fig. 2). Vaccine developments have been coupled with significant advances in our understanding of tumour DC biology within the last decade, but this increased knowledge also leads to more questions: which DC populations orchestrate responses to mRNA vaccines, and do they need to be directly transfected by vaccine antigens; do we need different strategies to target priming or intra-tumoural DC populations; are all DCs beneficial for tumour regression? It is an exciting time as technical advances allow us to investigate and manipulate anti-tumour immunity with more and more finesse.

## Data Availability

Not applicable.

## Abbreviations

ALK: anaplastic lymphoma kinase
APCs: antigen presenting cells
DCs: dendritic cells
ICIs: immune checkpoint inhibitors
IFN-α: interferon-alpha
LNPs: lipid nanoparticles
mCRPC: metastatic castration resistant prostate cancer
NGS: next-generation sequencing
PAP: prostatic acid phosphatase
PBMCs: peripheral blood mononuclear cells
TAA: Tumour associated antigens
TLR: toll-like receptor
TMB: tumour mutational burden
TME: tumour microenvironment
TSAs: tumour-specific antigens
